# Post-traumatic Stress Disorder and Associated Factors 1 Year After the Beginning of the COVID-19 Pandemic Among Chinese Residents

**DOI:** 10.3389/fpsyt.2021.766127

**Published:** 2021-11-03

**Authors:** Xin Shen, Shijiao Yan, Heng Jiang, Hui Cao, Rowan Dowling, Jing Feng, Zihui Lei, Crystal Jingru Li, Xiaotong Han, Chuanzhu Lv, Yong Gan

**Affiliations:** ^1^Department of Social Medicine and Health Management, School of Public Health, Tongji Medical College, Huazhong University of Science and Technology, Wuhan, China; ^2^School of Public Health, Hainan Medical University, Haikou, China; ^3^Key Laboratory of Emergency and Trauma of Ministry of Education, Hainan Medical University, Haikou, China; ^4^Centre for Alcohol Policy Research, School of Psychology and Public Health, La Trobe University, Melbourne, VIC, Australia; ^5^Melbourne School of Population and Global Health, University of Melbourne, Melbourne, VIC, Australia; ^6^Department of Labor Economics and Management, Beijing Vocational College of Labour and Social Security, Beijing, China; ^7^Department of Psychology, School of Education and Human Development, Hong Kong Education University, Hong Kong, China; ^8^Department of Emergency Medicine, Hunan Provincial Institute of Emergency Medicine, Hunan Provincial Key Laboratory of Emergency and Critical Care Metabolomics, Hunan Provincial People's Hospital/The First Affiliated Hospital, Hunan Normal University, Changsha, China; ^9^Emergency Medicine Centre, Sichuan Provincial People's Hospital, University of Electronic Science and Technology of China, Chengdu, China; ^10^Research Unit of Island Emergency Medicine, Chinese Academy of Medical Sciences, Hainan Medical University, Haikou, China

**Keywords:** PTSD, acute infectious diseases, long-term impact, COVID-19, low transmission period

## Abstract

**Background:** By investigating the incidence of post-traumatic stress disorder (PTSD) among residents during a period of low transmission, this study reflects the long-term impact of coronavirus disease 2019 (COVID-19) and identify which categories of residents are more likely to develop PTSD due to an acute infectious disease crisis, facilitating the development of targeted strategies to protect mental health after outbreaks of similar acute infectious diseases in the future.

**Methods:** A cross-sectional survey was conducted in China from 4 to 26 February 2021. A convenience sampling strategy was adopted to recruit participants. Participants were asked to complete the PTSD Checklist for DSM-5 (PCL-5). A multivariable linear stepwise regression analysis model was used to identify which factors were associated with PTSD in residents of China.

**Results:** A total of 2,361 Chinese residents completed the questionnaire. The mean PCL-5 score for the respondents was 13.65 (SD = 8.66), with 219 (9.28%) patients having probable PTSD symptoms. Respondents who were female (β = 0.038), had a relative or friend who had contracted COVID-19 (β = 0.041), and had poor health (β = 0.184) had higher PCL-5 scores, while the population aged over 60 years (β = −0.063), who agreed that COVID-19 information was released in a timely manner (β = −0.347), who had experienced a relatively limited impact of COVID-19 on their life (β = −0.069), and who agreed that the local prevention initiatives were sophisticated (β = −0.165) had lower PTSD scores.

**Conclusions:** Outbreaks of acute infectious diseases can have long-term psychological health effects in the general population. In addition, health policy makers need to be concerned about and implement measures to support the mental health of vulnerable groups.

## Background

Post-traumatic stress disorder (PTSD) can develop after traumatic events outside the range of common human experience, such as violent physical assaults, torture, accidents, rape or natural disasters, and is characterized by a typical pattern of symptoms involving intrusive thoughts, the persistence of the trauma, the avoidance of relevant stimuli, emotional numbness and physiological hyperarousal (1). The coronavirus disease 2019 (COVID-19) epidemic in China was first identified in late December 2019, when clusters of cases of pneumonia of unknown etiology were observed (2). The Chinese Lunar New Year holiday, the start of which coincided with the emergence of COVID-19, is the most celebratory time of the year in China, and mass panic was triggered by the declaration that the virus could be transmitted among humans (3). Since the start of the outbreak, the Chinese government has been swift to respond, and 3 weeks after the start of the epidemic, on 23 January, Wuhan was put into a lockdown in an unprecedented attempt to slow the spread of the virus, and travel into and out of the area was restricted. Within days, the lockdown was extended to additional provinces and cities, affecting more than 50 million people in total. Many people stayed at home and socially isolated themselves to avoid being infected, leading to a “desperate plea” (4). There have also been reports of shortages of masks and health care equipment. The ongoing emerging infectious disease crisis has induced fear (5).

Several studies have explored the psychological effects of epidemics, such as those involving severe acute respiratory syndrome (SARS) and H1N1 influenza. Mak et al. (6) and Lam et al. (7) both reported that more than 40% of SARS survivors had experienced PTSD at some time during the outbreak. Meanwhile, those respondents who had been isolated, worked in high-risk workplaces such as SARS wards, or had friends or close relatives who contracted SARS were two to three times more likely to develop high levels of PTSD than those who were not exposed to the disease (8). Consequently, PTSD should be given more attention during the outbreak of COVID-19.

During the COVID-19 outbreak, researchers in the United States (9), Italy (10), and Spain (11) performed studies on the prevalence of PTSD in the residents of their countries and found that the occurrence of PTSD was significantly related to demographic characteristics, risk perception and other factors (12). Chinese researchers have also investigated PTSD in different groups. For example, Tang et al. investigated the level of PTSD among college students (13), and Bo et al. surveyed the prevalence of PTSD among COVID-19 patients (14). However, unlike individual-level traumatic events, the COVID-19 outbreak has been a continuing crisis experienced by every member of society. There is a wide range of profound psychosocial impacts at the individual, community, and international levels during outbreaks of emerging infectious diseases. However, no studies have been conducted to investigate whether PTSD continues to affect the population after the achievement of control over the spread of COVID-19.

Currently, although the epidemic in China has entered a period of low transmission, the prevalence of PTSD in the population affected by this acute infectious disease is not clear. By investigating the prevalence of PTSD among Chinese residents during a period of low transmission, this study can reflect the long-term impact of COVID-19 and identify which subgroups of residents are more likely to develop PTSD due to an acute infectious disease crisis, facilitating the implementation of targeted strategies to protect mental health after outbreaks of similar acute infectious diseases in the future. As an increasing number of countries enter periods of low transmission, it becomes important to investigate the prevalence of and risk factors for PTSD in the population at this stage of the infectious disease outbreak. In 2021, the Chinese New Year fell on 4–26 February. This study explored the prevalence of PTSD due to the acute infectious disease crisis in the Chinese population 1 year after the start of the COVID-19 outbreak and the long-term psychological impact of the COVID-19 epidemic on the population, thereby filling in the gaps in available research. The results can serve as a reference for physical and mental health care policy makers.

## Methods

### Ethics Statement

The protocol for this study was approved by the institutional review board of Tongji Medical College of Huazhong University of Science and Technology, Wuhan, China. All methods were performed in accordance with the relevant guidelines and regulations. Respondents were informed that their participation was voluntary, and consent was implied by the completion of the questionnaire.

### Study Participants and Survey Design

A cross-sectional survey was conducted in China from 4 to 26 February 2021. A convenience sampling strategy was adopted to recruit participants; the research team used WeChat (the most popular social media platform in China) to advertise the study and circulate the survey link to their network members. Network members were asked to distribute the survey invitation to all their contacts. We collect information on respondents through Questionstar, a popular survey distribution and collection site. The site will automatically identify and eliminate non-responders. Respondents were stratified according to the regions of China as follows: East (Beijing, Tianjin, Hebei, Liaoning, Shanghai, Jiangsu, Zhejiang, Fujian, Shandong, Guangdong and Hainan), Central (Shanxi, Jilin, Heilongjiang, Anhui, Jiangxi, Henan, Hubei, and Hunan) and Western (Chongqing, Sichuan, Guizhou, Yunnan, Tibet, Shaanxi, Gansu, Qinghai, Ningxia, Xinjiang, Inner Mongolia, and Guangxi). Participants were informed that their participation was voluntary, and their consent was implied by their completion of the questionnaire. The inclusion criteria were as follows: 1) Chinese citizens who were at least 18 years old and 2) able to comprehend and read Chinese.

In our study, a 95% confidence level and ±5% precision are assumed for the Equation.


$$\begin{array}{l} n=\frac{N}{1+N{\left(e\right)}^{2}} \end{array}$$

Where n is the sample size, N is the population size, and e is the level of precision. Thus, the conservative total sample size for this questionnaire is 1,200.

### Instruments

The survey consisted of questions that assessed the following: 1) the participants' demographic background; 2) the participants' perception of their personal risk during the COVID-19 pandemic (measuring exposure level is essential to understanding the implications of the prevalence of PTSD; this study measured “perceived exposure” based on whether the respondent had contracted COVID-19, whether they had lost relatives or acquaintances, the duration of lockdown, etc.); and 3) the PTSD Checklist for DSM-5 (PCL-5). Demographic information, including sex, age, marital status, place of residence, highest education level attained, region, and employment status, was collected. The assessment of the participants' personal risk perception during the COVID-19 pandemic gathered information on participants' experiences during, perceptions of, and attitudes toward the COVID-19 pandemic.

PTSD was assessed with the PCL-5 (15), which was compiled by Bragesjö et al. (16) and translated into Chinese and revised by Zhou et al. (17). A total of 20 items rated on a five-point scale ranging from 0 (never) to four (severe) were used to assess the frequency of symptoms after being diagnosed with COVID-19. The participants were asked to report their symptoms in the last month. The PCL-5 is composed of four dimensions: intrusion, emotion alteration, avoidance, and hyperarousal. A total score is computed for each dimension, with higher scores indicating a higher degree of PTSD symptoms. Based on the clinical criteria (18), scores >33 indicate PTSD symptoms. The psychometric properties of Chinese version of the original PCL-5 have recently been validated, and it has been widely used in trauma-related research and practice (19). In the current Chinese study, Cronbach's alpha for the scale was 0.962 (20).

China was the first country to be affected by the COVID-19 outbreak and was ultimately severely affected, resulting in a high risk of infection among the residents. According to the Life Events Checklist for DSM-5 (LEC-5), which is part of the PCL-5, this type of experience constitutes a traumatic event (21). Thus, in the present study, residents of different regions of China were selected and instructed to rate how much they were affected by the outbreak of COVID-19 in the last month by completing the PCL-5. The PCL-5 not only reflects the degree of PTSD symptoms experienced by an individual but can also be used to measure the prevalence of PTSD in a population based on a cut-off score. Even in a population of residents not clinically diagnosed with PTSD, there are significant differences in PCL-5 scores. Therefore, this study used this scale to identify which groups were more likely to have PTSD. Using the PCL-5 score as the dependent variable enables the exploration of the subtle differences among various groups of people, facilitating the identification of the groups at risk for poor mental health who need targeted support.

### Statistical Methods

Descriptive analysis included the calculation of the means and standard deviations of continuous variables and the numbers and percentages of categorical variables. *T*-tests and ANOVA were used to compare factors affecting PTSD between residents in different regions. No clustering was observed in the respondents (correlation = 0.03, *P* < 0.001). Therefore, a multivariable linear stepwise regression analysis model was used to identify the factors associated with PTSD in the respondents (inclusion and exclusion criteria were *P* = 0.05 and *P* = 0.01, respectively). For each comparison, the *P* values were corrected for multiple comparisons to control the false discovery rate. We used a variance inflation factor to assess multicollinearity. All analyses were performed using STATA 12.0, and all differences were tested with two-tailed tests. A *P* < 0.05 was considered statistically significant.

## Results

### Descriptive Statistics

A total of 2,453 residents received the questionnaire. The response rate was 96.24%, with 21 participants who did not respond and 71 questionnaires that were not completed. The remaining 2,361 complete questionnaires were used in our analysis. Table 1 reports the socio-demographic characteristics of the 2,361 respondents. The mean age was 29.72 years (18–77, SD = 6.94), and the majority of respondents were female (60.10%). Among the respondents, 421 (17.83%), 1,470 (62.26%), and 470 (19.91%) were from East, Central, and Western China, respectively. Most respondents (89.24%) reported having attained a bachelor's degree or higher. More than half of the participants were unemployed (57.05%), unmarried (66.07%) and lived in urban areas (58.11%).

**Table 1:** Statistical description of study samples.

**Variables**	***N* (%)**	**PCL-5 Scores**	***F/t***	***P-*value**
		**M (SD)**		
Total	2,361 (100)	19.65 (12.66)	NA	NA
**Gender**				
Male	942 (39.90)	20.16 (13.00)	2.60	0.11
Female	1,419 (61.10)	19.30 (12.41)		
**Age group, y**				
18–44	1,845 (78.14)	20.36 (12.81)	18.75	<0.001
45–59	369 (15.63)	18.18 (12.23)		
>60	111(4.70)	14.32 (9.93)		
**Marital status**				
Unmarried	1,560 (66.07)	20.24 (12.94)	10.261	0.001
Married	801 (33.93)	18.48 (11.99)		
**Place of residence**				
Urban	1,372 (58.11)	18.91 (12.21)	11.282	0.001
Rural	989 (41.89)	20.67 (13.18)		
**Highest educational level attained**				
Primary school or below	68 (2.88)	21.87 (13.85)	3.014	0.049
middle school	186 (7.88)	21.33 (12.09)		
College degree or above	2,107 (89.24)	19.43 (12.65)		
**Region**				
Eastern China	421 (17.83)	19.32 (12.55)	1.498	0.224
Central China	1,470 (62.26)	19.45 (12.76)		
Western China	470 (19.91)	20.54 (12.41)		
**Employment status**				
Employed	1,014 (42.95)	19.33 (12.70)	1.129	0.288
Unemployed	1,347 (57.05)	19.89 (12.62)		
**Relative or friend has experienced COVID-19**				
Yes	206 (8.73)	19.46 (12.52)	5.161	0.023
No	2,155 (91.27)	21.56 (13.90)		
**Consent COVID-19 information has been released timely**				
Yes	657 (27.83)	27.06 (12.05)	360.395	<0.001
No	1,704 (72.17)	16.79 (11.69)		
**COVID-19 have a low impact on your life**				
Strongly disagree	620 (26.26)	21.13 (12.99)	4.328	0.002
Disagree	964 (40.83)	19.70 (12.66)		
Not sure	562 (23.80)	18.10 (11.97)		
Agree	124 (5.25)	17.26 (11.10)		
Strongly agree	91 (3.85)	18.64 (12.51)		
**Individual health is currently poor**				
Strongly disagree	65 (2.75)	20.40 (12.61)	0.728	0.572
Disagree	67 (2.84)	21.48 (12.70)		
Not sure	436 (18.47)	18.98 (12.69)		
Agree	955 (40.45)	19.74 (12.61)		
Strongly agree	838 (35.49)	19.68 (12.70)		
**Keeping concerning health information**				
Strongly disagree	74 (3.13)	21.36 (13.63)	3.488	0.008
Disagree	179 (7.58)	21.17 (13.10)		
Not sure	752 (31.85)	20.51 (13.01)		
Agree	815 (34.52)	19.20 (12.38)		
Strongly agree	541 (22.91)	18.38 (12.16)		
**The local prevention initiatives are sophisticated**				
Strongly disagree	66 (2.80)	22.03 (14.03)	1.175	0.320
Disagree	136 (5.76)	19.80 (13.33)		
Not sure	691 (29.27)	20.13 (12.66)		
Agree	890 (37.30)	19.43 (12.31)		
Strongly agree	578 (24.48)	19.10 (12.84)		

The mean score on the PCL-5 was 13.65 (SD = 8.66). Using a cut-off score of 33, 219 (9.28%) patients had probable PTSD symptoms. Table 1 shows the mean scores in different subpopulations. There were significant differences in PCL-5 scores based on age, marital status, place of residence, highest education level attained, presence of relatives or friends with COVID-19, agreement that information about COVID-19 has been released in a timely manner, agreement that COVID-19 has had a limited impact on their lives, and “keeping concerning health information”.

Table 2 lists the results of the multivariable linear stepwise regression analysis of the factors associated with PTSD. Female sex (β = 0.038), relative or friend with COVID-19 (β = 0.041), and poor health (β = 0.184) were associated with higher PCL-5 scores, while age > 60 years (β = −0.063), agreement that information about COVID-19 has been released in a timely manner (β = −0.347), perception that COVID-19 had a limited impact on their life (β = −0.069), and agreement that the local prevention initiatives were sophisticated (β = −0.165) were associated with lower PCL-5 scores.

**Table 2:** Stepwise regression analysis of associated factors for PTSD among respondents.

**Variables**	**Unstandardized coefficients**		**Standardized coefficients**	***t***	***p*-value**	**95%CI**
	**β**	***SE***	**β**			
Total	19.37	2.38	NA	8.138	<0.001	14.963 ~ 24.272
Gender (Ref: male)						
Female	0.99	0.497	0.038	1.994	0.046	0.006 ~ 1.947
Age group, y (Ref: 18–44)						
45–59	−1.08	0.677	−0.031	−1.596	0.111	−2.446 ~ 0.200
>60	−3.30	1.031	−0.063	−3.199	0.001	−5.278 ~−1.245
Marital status (Ref: unmarried)						
Married	−2.58	0.701	−0.097	−3.685	<0.001	−3.955 ~−1.209
Place of residence (Ref: urban)						
Rural	−0.636	0.548	−0.025	−1.161	0.246	−1.710 ~ 0.438
Highest educational level attained (Ref: primary school or below)						
Middle school	0.687	1.699	0.015	0.404	0.686	−2.644 ~ 4.018
College degree or above	−0.545	1.495	−0.013	−0.365	0.715	−3.475 ~ 2.385
Region (Ref: Eastern China)						
Central China	0.706	0.669	0.027	1.054	0.292	−0.606 ~ 2.018
Western China	0.951	0.792	0.03	1.202	0.230	−0.600 ~ 2.503
Employment status (Ref: employed)						
Unemployed	0.897	0.687	0.035	1.306	0.192	−0.449 ~ 2.243
Relative or friend has experienced COVID-19 (Ref: no)						
Yes	1.825	0.869	0.041	2.1	0.036	0.122 ~ 3.528
Consent COVID-19 information has been released timely (Ref: no)						
Yes	−9.803	0.556	−0.347	−17.625	<0.001	−10.893 ~−8.713
COVID-19 have a low impact on your life (Ref: strongly disagree)						
Disagree	−1.104	0.606	−0.043	−1.821	0.069	−2.292 ~ 0.084
Not sure	−1.91	1.158	−0.034	−1.649	0.099	−4.181 ~ 0.360
Agree	−2.259	1.319	−0.034	−1.713	0.087	−4.843 ~ 0.326
Strongly agree	−2.053	0.684	−0.069	−3.000	0.003	−3.394 ~−0.712
Individual health is currently poor (Ref: strongly disagree)						
Disagree	3.954	2.562	0.052	1.543	0.123	−1.068 ~ 8.975
Not sure	2.644	2.309	0.081	1.145	0.252	−1.881 ~ 7.169
Agree	3.903	2.275	0.151	1.715	0.086	−0.557 ~ 8.362
Strongly agree	4.867	2.29	0.184	2.125	0.034	0.379 ~ 9.354
Keeping concerning health information (Ref: strongly disagree)						
Disagree	0.44	1.877	0.009	0.234	0.815	−3.240 ~ 4.119
Not sure	0.022	1.718	0.001	0.013	0.99	−3.346 ~ 3.389
Agree	−0.984	1.716	−0.037	−0.574	0.566	−4.347 ~ 2.379
Strongly agree	−1.639	1.743	−0.054	−0.94	0.347	−5.056 ~ 1.778
The local prevention initiatives are sophisticated (Ref: strongly disagree)						
Disagree	−4.104	2.475	−0.076	−1.658	0.097	−8.956 ~ 0.748
Not sure	−3.979	2.353	−0.143	−1.691	0.091	−8.590 ~ 0.632
Agree	−4.567	2.343	−0.175	−1.949	0.051	−9.160 ~ 0.026
Strongly agree	−4.851	2.389	−0.165	−2.03	0.042	−9.533 ~−0.168

Table 3 reports the stratified results of the multivariable linear regression of the predictions regarding PTSD by region. There were differences in the predicted results regarding psychological resilience among regions. In East China, residents who agreed that information about COVID-19 had been released in a timely manner (β = −0.409) had lower PCL-5 scores. In Central China, sex (β = 0.062), agreement that information about COVID-19 had been released in a timely manner (β = −0.317) and perception that COVID-19 had a limited impact on their life (β = −0.074) were significantly associated with the PCL-5 scores. In Western China, age > 60 years (β = −0.096) and agreement that information about COVID-19 had been released in a timely manner (β = −0.333) were associated with lower PCL-5 scores.

**Table 3:** Stratified stepwise regression of predictors of psychological resilience by region.

**Variables**	**Unstandardized coefficients**		**Standardized coefficients**	***t***	***p*-value**	**95%CI**
	**β**	***SE***	**β**			
Total	19.37	2.38	–	8.138	<0.001	14.963 ~ 24.272
**Eastern China**						
Consent COVID-19 information has been released timely (Ref: no)						
Yes	−10.804	1.243	−0.409	−8.692	<0.001	−13.240 ~−8.368
**Central China**						
Gender (Ref: male)						
Female	1.618	0.650	0.062	2.488	0.013	0.344 ~ 2.893
Consent COVID-19 information has been released timely (Ref: no)						
Yes	−9.520	0.752	−0.317	−12.658	<0.001	−10.994 ~−8.046
COVID-19 have a low impact on your life (Ref: strongly disagree)						
Strongly agree	−2.228	0.892	−0.074	−2.498	0.013	−3.976 ~−0.480
**Western China**						
Age group, y (Ref: 18–44)						
>60	−3.957	1.913	−0.096	−2.069	0.039	−7.705 ~−0.208
Consent COVID-19 information has been released timely (Ref: no)						
Yes	−8.628	1.229	−0.333	−7.022	<0.001	−11.037 ~−6.220

## Discussion

We firstly evaluate the consequences of the COVID-19 outbreak with regard to PTSD ~ 1 year after the start of the outbreak in China and identify the related risk factors. The mean psychological resilience score of the respondents was 13.65 (SD = 8.66), and 219 (9.28%) participants had higher PCL-5 scores. Sex, age, relative or friend with COVID-19, poor health, agreement that information about COVID-19 had been released in a timely manner, perception that COVID-19 had a limited impact on their life and agreement that local prevention initiatives were sophisticated were the main factors associated with the residents' PCL-5 scores.

The COVID-19 pandemic is a significant source of psychological stress and has had tremendous impacts on all facets of individuals' lives and organizations' operations in virtually every social and economic sector worldwide. Fear of illness and uncertainty about the future can lead to the development of anxiety- and stress-related disorders. Fear of illness, fear of death, and uncertainty regarding the future are significant psychological stressors, and social isolation resulting from the loss of structured education and work activities also threatens to worsen public mental health. The world has been affected by an unprecedented pandemic remains poorly understood. Previous studies on COVID-19 have reported a prevalence of PTSD-related symptoms of ~ 5% in Wuhan, the first area in China affected by the COVID-19 epidemic. This result was observed by administering the PCL-5 and by using a cut-off score of 33 points (22). Our study found that many people were still have higher PCL-5 scores nationwide even a year after the start of the pandemic.

Female respondents were found to have more intrusive thoughts than males. This result is consistent with previous studies, which showed that after traumatic events, acute psychological disorders characterized by intrusive memories are more prevalent in women than men (23, 24). Some evidence suggests that fluctuations in ovarian hormone levels are responsible for differences in sensitivity to emotional stimuli during certain phases in the menstrual cycle, during which intrusive flashbacks are enhanced, possibly explaining the increased vulnerability of women to psychological disorders (25). Similarly, age and marital status were important factors affecting PCL-5 scores. Several studies have suggested that demographic characteristics are critical factors affecting the predisposition to PTSD (10, 10, 13, 26). Our study also shows that older adults are more likely to get higher PCL-5 scores.

During one influenza outbreak, ~ 10–30% of the general public was very or fairly worried about the possibility of contracting the virus (27). With the closure of schools and business, the negative emotions experienced by individuals can be compounded (27). During the SARS outbreak, many studies investigated the psychological impact on the uninfected community and showed that there were significant psychiatric morbiditie associated with younger age and increased self-blame (28). In our study, residents who had relatives or friends who contracted COVID-19, had poor health, and experienced a large impact due to COVID-19 had higher PCL-5 scores, indicating that vulnerable groups are more likely to have psychological problems. Residents who agreed that information about COVID-19 information had been released in a timely manner and that local prevention initiatives were sophisticated had lower PCL-5 scores, which showed that government policies have significant impacts on the mental health of residents. Therefore, efforts need to be made to support residents and break the vicious circle of fear and panic related to COVID-19. A combination of strategies is required at both the public and individual levels, which could include public engagement, support for individual self-adjustment through participation in stress-releasing activities, familial support, and even psychiatric help (29).

Addressing PTSD requires a multifaceted approach. Individuals should assess the COVID-19 situation objectively, determine the risk they face in different situations, and take scientific protective measures to reduce their fears and inappropriate protective behaviors. They should also recognize and overcome their PTSD emotions. When individuals are unable to cope with PTSD, seek help from health care agencies or the government. The government should take the initiative to understand the mental health and PTSD status of the population, identify high-risk groups in a timely manner, and avoid suicide, impulsive behavior and extreme events. Medical institutions shall carry out publicity and education and provide mental health services.

### Strengths and Limitations

This is the first study to measure PTSD in the population during a period of low transmission, showing the long-term psychological damage caused by the COVID-19 outbreak in the general population. Meanwhile, we investigated the major factors influencing the psychological health of residents 1 year after the start of the COVID-19 outbreak and identified vulnerable populations in need of mental health support. We involved a nationwide sample of the Chinese population, and the results could be useful to countries entering a period of low transmission.

However, this study has some limitations. First, we used social media as the main method of disseminating the survey. Participants without access to the internet were probably not included. In the absence of data regarding epidemiological variables (e.g., age, education, employment etc.) for the participants, it is difficult to determine the representativeness of the sample. Second, the distribution of the study participants was imbalanced across regions (421: 1,470: 470); therefore, the sample might not be representative of the national population. In addition, under convenience sampling, more female, younger age, and educated participants were also brought. Future studies need to be more detailed, based on population and geography. Third, we could not determine how many participants viewed the online poster or survey but decided not to complete the survey; thus, the presence of non-response bias could not be assessed. Finally, as the behaviors were self-reported, reporting bias was possible. Overall, generalization of the results should be performed with caution.

## Conclusions

In a period of low transmission of COVID-19, the mean PCL-5 score among Chinese respondents was 13.65 (SD = 8.66), and 219 (9.28%) participants had probable PTSD symptoms. This means that outbreaks of acute infectious diseases can have long-term psychological health effects in the general population. In addition, health care policy makers need to be concerned about and support the mental health of vulnerable groups.

## Data Availability Statement

The raw data supporting the conclusions of this article will be made available by the authors, without undue reservation.

## Ethics Statement

All individuals provided written informed consent. This study was performed in line with the principles of the Declaration of Helsinki. The protocol strictly abided by the Chinese Statistical Law to ensure that participants' personal information was kept confidential. This study protocol was approved by the institutional review board of Tongji Medical College of Huazhong University of Science and Technology, Wuhan, China ([2021]S088).

## Author Contributions

XS, SY, and YG: conceived and designed the study. JF and ZL: participated in the acquisition of data. HC and HJ: analyzed the data. RD and CJL: gave advice on methodology. XS and SY: drafted the manuscript. XH, CL, and YG: revised the manuscript. YG is the guarantor of this work and had full access to all the data in the study and takes responsibility for its integrity and the accuracy of the data analysis. All authors have read and approved the final manuscript.

## Funding

This study was supported by the Fundamental Research Funds for the Central Universities (2020kfyXJJS059), Hainan Provincial Key Research and Development Project (ZDYF2020112), and National Natural Science Foundation of China (82160647).

## Conflict of Interest

The authors declare that the research was conducted in the absence of any commercial or financial relationships that could be construed as a potential conflict of interest.

## Publisher's Note

All claims expressed in this article are solely those of the authors and do not necessarily represent those of their affiliated organizations, or those of the publisher, the editors and the reviewers. Any product that may be evaluated in this article, or claim that may be made by its manufacturer, is not guaranteed or endorsed by the publisher.
